# Early-life nutritional deprivation and multimorbidity in middle- aged and older adults: a longitudinal exploratory mediation analysis of biological aging

**DOI:** 10.3389/fnut.2026.1908542

**Published:** 2026-09-10

**Authors:** Qiaohe Wang, Li Ma, Zhuo Tian, Qiulin Luo, Huamin Liao

**Affiliations:** 1 Department of Special Medicine, Chongqing Academy of Medical Sciences, Chongqing General Hospital, Chongqing University, Chongqing, China; 2 Department of Psychosomatic Medicine, Xinjiang Production and Construction Corps 13th Division Red Star Hospital, Hami, China

**Keywords:** biological age, causal mediation analysis, CHARLS, childhood food deprivation, Light-BioAge, PPC-MM

## Abstract

**Objective:**

This study aimed to examine the longitudinal association between childhood food deprivation and physical-psychological-cognitive multimorbidity (PPC-MM), and to explore whether Light-BioAge mediates this association.

**Methods:**

This study included 4546 participants aged ≥45 years from the China Health and Retirement Longitudinal Study (CHARLS). Childhood food deprivation was retrospectively assessed as none, moderate, or extreme. Incident PPC-MM was identified during 2011–2020 follow-up. Cox regression examined the longitudinal association, and causal mediation analysis explored the potential mediating effect of Light-BioAge.

**Results:**

In the unadjusted model, compared with no deprivation, moderate deprivation was associated with a 20% increased risk of PPC-MM (HR = 1.20, 95% CI: 1.10–1.31), and extreme deprivation with a 60% increased risk (HR = 1.60, 95% CI: 1.40–1.82). After adjusting for socio-demographic factors, the HRs were 1.19 (95% CI: 1.08–1.30) and 1.44 (95% CI: 1.26–1.65), respectively. Further adjustment for lifestyle and health-related covariates yielded HRs of 1.17 (95% CI: 1.07–1.28) and 1.43 (95% CI: 1.25–1.63). Kaplan-Meier survival curves showed significant differences in cumulative incidence among the three groups (Log-rank test, *P* < 0.001). Subgroup analyses revealed consistent directionality across sex, age, education, residence, and BMI strata, with no significant interactions (all P for interaction > 0.05). Exploratory causal mediation analysis indicated that Light-BioAge accounted for a small proportion (4.9% and 5.3%) of the total effects, with indirect effects close to the null (HR = 1.01, 95% CI: 1.00–1.01).

**Conclusion:**

Childhood food deprivation is robustly associated with PPC-MM in a dose-response manner. The exploratory effect of Light-BioAge suggests that other pathways may play more important roles. These findings support integrating early-life nutritional risk assessment into healthy aging strategies.

## Background

With the extension of global life expectancy and the increasing burden of chronic non-communicable diseases, multimorbidity has become increasingly prevalent, affecting over 50% of older adults (1, 2). Multimorbidity not only leads to functional decline, reduced quality of life, and increased healthcare utilization, but is also closely associated with elevated premature mortality risk (3, 4). Physical-psychological-cognitive multimorbidity (PPC-MM), the intertwined state of chronic physical diseases, mental disorders, and cognitive impairment, represents a key manifestation of multimorbidity, with prevalence varying significantly across populations and regions (5). This cross-dimensional health deterioration reflects profound systemic decompensation in physiological regulation and psychosocial adaptation (6). Given the dual burden of PPC-MM on individual health outcomes and socioeconomic costs, identifying its modifiable early-life risk factors has become central to advancing healthy aging strategies (3, 7).

Early-life exposures have increasingly been recognized as critical upstream determinants shaping late-life health trajectories. Existing research has shown that childhood adversity (such as poor health status or socioeconomic disadvantage) is an important factor predicting susceptibility to multimorbidity in later life (1, 8) and this influence exhibits long-lasting and relatively independent properties, meaning that even improvements in adult socioeconomic status or optimization of health behaviors cannot fully erase the biological imprint formed during childhood (8, 9). Childhood adversity is independently associated with higher frailty risk in middle-aged and older adults (10), more unfavorable trajectories of depressive symptoms (11–13), and cognitive decline (14, 15). Among various childhood adversities, childhood food deprivation represents a typical experience of material deprivation, directly affecting nutrition and stress systems during critical developmental periods. Current literature has separately demonstrated that early-life food deprivation relates to frailty risk in middle-aged and older adults (16), as well as with overweight, difficulties in activities of daily living, depressive symptoms, and cognitive decline (17, 18). However, these associations have been limited to isolated analyses of single health outcomes, and childhood food deprivation has not yet been examined within the integrated framework of physical-psychological-cognitive multimorbidity.

Studies have shown that childhood adverse experiences are associated with telomere shortening (19), advanced DNA methylation age (20), and elevated inflammatory markers (21), suggesting that early stress may influence long-term health through cellular senescence pathways. Quantifying the degree of such aging acceleration requires reliable biological indicators. Compared with chronological age, biological age can more comprehensively reflect the actual physiological functional status and overall health level of multiple body systems (22). Light-BioAge, as a simplified derivative indicator derived from the Gompertz law of mortality, is calculated by integrating three clinically routine parameters: serum creatinine, blood glucose, and log-transformed C-reactive protein, providing a practical and efficient tool for quantifying individual physiological wear and aging speed in large-scale cohort studies. This model has been validated in multiple independent cohorts for its significant association with health outcomes (22). Yet, existing research has not yet utilized this tool to examine the mediating effect of biological aging in the association between childhood food deprivation and PPC-MM.

Therefore, based on the CHARLS database, this study primarily aims to examine the longitudinal dose-response association between childhood food deprivation and PPC-MM incidence risk, and to explore whether Light-BioAge is associated with this pathway. By establishing this association, this study expects to provide preliminary evidence for incorporating early-life nutritional risk assessment in healthy aging strategies.

## Methods

### Data source and study population

The data were obtained from the CHARLS, a nationally representative cohort of Chinese adults aged ≥45 years. CHARLS employed multistage stratified probability-proportionate-to-size sampling and conducted baseline surveys in 28 provinces in 2011, with biennial follow-ups thereafter. Data were collected through face-to-face interviews using structured questionnaires, with individual weights to ensure national representativeness. The study was approved by the Peking University Ethics Committee (IRB00001052-11015), with written informed consent from all participants. Further details on CHARLS design are available elsewhere (23).

**FIGURE 1:**
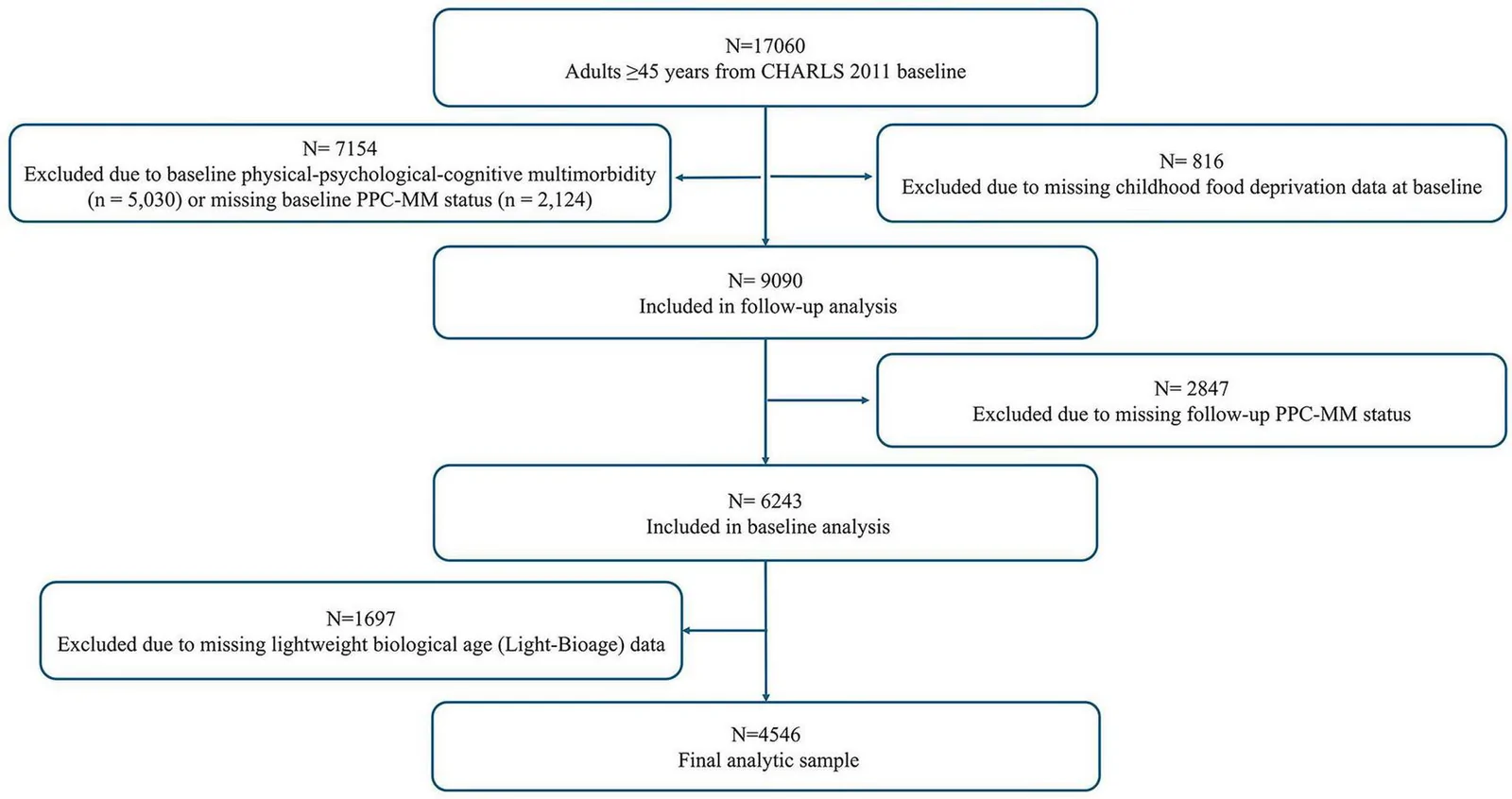
Diagram showing the participants included and excluded from the study.

This study included participants aged ≥45 years from the 2011 CHARLS baseline survey who were free of PPC-MM at baseline. Childhood food deprivation was retrospectively assessed through the 2014 life history survey, which evaluated food sufficiency before age 12. Eligibility for analysis required: (1) complete childhood food deprivation information; (2) non-missing biomarker data for serum creatinine, fasting glucose, and C-reactive protein at baseline to enable Light-BioAge calculation; and (3) complete follow-up PPC-MM assessment data through 2020. Of the 17,060 adults from the 2011 baseline, 7,154 were excluded due to prevalent baseline PPC-MM or missing baseline PPC-MM status, and 816 were excluded due to missing childhood food deprivation data, yielding 9,090 participants for longitudinal analysis. Subsequently, 2,847 participants were excluded due to loss to follow-up or missing PPC-MM status during the follow-up period, leaving 6,243 with complete follow-up data. Finally, 1,697 were excluded due to missing baseline Light-BioAge data, resulting in a final analytic sample of 4,546 participants (Figure 1).

### Exposure–childhood food deprivation

Information on childhood food deprivation was collected from the CHARLS 2014 life history survey. Following established definitions, participants were classified into three ordered categories:

No deprivation: Participants who did not report food insufficiency before age 12. Moderate deprivation: Participants who self-reported insufficient food to eat before age 12. Extreme deprivation: Participants who met both of the following criteria: self-reported food insufficiency before age 12; and either born and raised in documented famine-affected areas during specific historical famine periods, or born during the Great Famine (1958–1962) with reported severe family-level consequences (e.g., immediate family members or siblings starved to death, delayed conception, or abortion) (16).

### Outcome – physical-psychological-cognitive multimorbidity

Physical-psychological-cognitive multimorbidity was operationalized as a binary outcome. Participants were classified as having PPC-MM if they concurrently met criteria for at least two of the three domains (physical, psychological, and cognitive), encompassing four specific patterns: physical-psychological multimorbidity, physical-cognitive multimorbidity, psychological-cognitive multimorbidity, and physical-psychological-cognitive multimorbidity (24).

Physical conditions were identified by self-reported physician diagnosis of any of the following seven chronic diseases: hypertension, diabetes, cancer, chronic lung disease, heart disease, stroke, or arthritis (25, 26).

Psychological condition was assessed using the 10-item Center for Epidemiological Studies Depression Scale (CESD-10), with a score of ≥ 10 indicating the presence of depression (27).

Cognitive condition was evaluated through a battery of cognitive tests encompassing three domains: episodic memory (immediate and delayed recalls), orientation (recall of time, place, and person), and executive function. These tests have been validated as feasible and effective in the Chinese population, showing significant correlations with the Clinical Dementia Rating (CDR) scale (28). Cognitive impairment was defined as performance > 1.5 standard deviations below age-group-specific means in at least one domain (24).

### Mediating factors

Light-BioAge, a simplified derivative of the Gompertz Law-Based Biological Age (GOLD BioAge) developed by the Fudan University research team, was computed using the following equation (22): *BioAge* = *Age* + 8.3313 × *Creatinine* + 0.8270 × *Glucose* + 5.7305 × *LogCRP* − 13.5298 where Age is chronological age (years), Creatinine is serum creatinine (mg/dL), Glucose is fasting glucose (mmol/L), and LogCRP is log-transformed C-reactive protein (mg/L). All four variables were acquired via questionnaire interview and laboratory testing. The model was validated across three independent Chinese older adult cohorts (CHARLS, RuLAS, and CLHLS), confirming its predictive validity for mortality risk and demonstrating a significant association with frailty onset (22).

### Other covariates

Referring to the results of previous studies (16, 22, 24), this research included socio-demographic, family background, socioeconomic, and health and lifestyle covariates. Socio-demographic covariates included age group (45–59 years or ≥60 years), gender (female or male), ethnicity (Han Chinese or ethnic minorities), and residence (urban or rural). Marital status was classified as married or other marital statuses (divorced, widowed, or never married). Education level was categorized as illiterate, primary school, middle school, or high school and above. Family background variables included maternal agricultural work before age 17 (yes or no) and paternal agricultural work before age 17 (yes or no). Socioeconomic covariates included pension insurance (yes or no), medical insurance (yes or no), and social participation (yes or no). Health and lifestyle covariates included body mass index (BMI, kg/m^2^), smoking status (never smoker, former smoker, or current smoker), and drinking status (never drinker, former drinker, or current drinker).

### Statistical analyses

Data processing and analysis were performed using R version 4.4.0, with a two-sided *P*-value < 0.05 considered statistically significant. Participants were described according to the total sample and childhood food deprivation groups (no deprivation, moderate deprivation, extreme deprivation), with continuous variables presented as median (Q_1_, Q_3_) and categorical variables as frequencies (percentages); group differences were assessed using Kruskal–Wallis tests and Chi-square tests, respectively. The cumulative incidence of PPC-MM across childhood food deprivation groups was visualized using Kaplan-Meier curves with log-rank tests. Cox proportional hazards regression models were used to estimate hazard ratios (HRs) and 95% confidence intervals (CIs) for the association between childhood food deprivation and incident PPC-MM, with three models constructed: Model 1 unadjusted; Model 2 adjusted for age, sex, marital status, ethnicity, residence and education level; and Model 3 fully adjusted for age, sex, residence, ethnicity, education level, marital status, maternal agricultural work before age 17, paternal agricultural work before age 17, pension insurance, medical insurance, social participation, smoking status, drinking status, and BMI. The proportional hazards assumption was examined using Schoenfeld residual tests; no significant violations were observed (global test: χ^2^ = 19.260, df = 20, *P* = 0.505; childhood food deprivation: χ^2^ = 0.523, df = 2, *P* = 0.770; Supplementary Table 3). Multicollinearity was assessed by generalized variance inflation factors (GVIF); no severe collinearity was detected (all GVIF∧ (1/2Df) < 1.5, well below the threshold of 10; Supplementary Table 2). Stratified analyses were conducted by age group (45–59 vs. ≥60 years), gender, residence, with interaction terms between childhood food deprivation and each stratification variable tested in the fully adjusted Cox model and results presented as forest plots. Mediation analyses were conducted using the R package regmedint (v1.0.2) (29). Cox proportional hazards models were used for the time-to-event outcome and linear regression for the mediator. Natural direct and indirect effects were estimated on the log-hazard ratio scale, evaluated at mean covariate values. Missing data imputation was performed using linear and logistic regression models, with final imputed values derived through nearest neighbor matching based on the predictive mean matching (PMM) framework.

## Results

### Participants’ characteristics

A total of 4546 participants were included in this study, including 1828 (40.21%) with no childhood food deprivation, 2211 (48.64%) with moderate childhood food deprivation, and 507 (11.15%) with extreme childhood food deprivation. The median BMI was 23.63 (21.35–26.05) kg/m^2^, and 36.05% of the participants were aged 60 years or older. Males accounted for 51.17%, and ethnic minorities accounted for 6.67%. Significant differences across the three groups were observed in age, marital status, maternal occupation, paternal occupation, pension insurance, sex, ethnic minority status, residence, education, smoking status, drinking status, and age group (all *P* < 0.05). No significant differences were found in BMI, medical insurance, or social activity (all *P* > 0.05). Details are shown in Table 1.

**TABLE 1:** Baseline characteristics by food deprivation status.

Variables	Total (*n* = 4546)	No FD (*n* = 1828)	Moderate FD (*n* = 2211)	Extreme FD (*n* = 507)	Statistic	*P*
BMI (kg/m^2^), M (Q_1_, Q_3_)	23.63 (21.35, 26.05)	23.65 (21.21, 26.05)	23.68 (21.49, 26.14)	23.34 (21.38, 25.54)	χ^2^ = 2.73^#^	0.255
Age group, n (%)					χ^2^ = 49.68	**<0.001**
45–59	2907 (63.95)	1150 (62.91)	1497 (67.71)	260 (51.28)		
≥60	1639 (36.05)	678 (37.09)	714 (32.29)	247 (48.72)		
Sex, n (%)					χ^2^ = 32.89	<0.001
Female	2220 (48.83)	986 (53.94)	1014 (45.86)	220 (43.39)		
Male	2326 (51.17)	842 (46.06)	1197 (54.14)	287 (56.61)		
Ethnicity, n (%)					χ^2^ = 17.30	**<0.001**
Han Chinese	4243 (93.33)	1701 (93.05)	2047 (92.58)	495 (97.63)		
Ethnic minorities	303 (6.67)	127 (6.95)	164 (7.42)	12 (2.37)		
Residence, n (%)					χ^2^ = 8.91	**0.012**
Urban	1726 (37.97)	727 (39.77)	834 (37.72)	165 (32.54)		
Rural	2820 (62.03)	1101 (60.23)	1377 (62.28)	342 (67.46)		
Marital status, n (%)					χ^2^ = 10.96	**0.004**
Married	341 (7.50)	163 (8.92)	137 (6.20)	41 (8.09)		
Other marital statuses	4205 (92.50)	1665 (91.08)	2074 (93.80)	466 (91.91)		
Education level, n (%)					χ^2^ = 64.14	**<0.001**
Illiterate	1795 (39.49)	654 (35.78)	883 (39.94)	258 (50.89)		
Primary school	1071 (23.56)	400 (21.88)	547 (24.74)	124 (24.46)		
Middle school	1081 (23.78)	498 (27.24)	498 (22.52)	85 (16.77)		
High school and above	599 (13.18)	276 (15.10)	283 (12.80)	40 (7.89)		
Maternal agricultural work before age 17, n (%)					χ^2^ = 25.27	**<0.001**
No	310 (6.82)	161 (8.81)	134 (6.06)	15 (2.96)		
Yes	4236 (93.18)	1667 (91.19)	2077 (93.94)	492 (97.04)		
Paternal agricultural work before age 17, n (%)					χ^2^ = 30.36	**<0.001**
No	906 (19.93)	432 (23.63)	402 (18.18)	72 (14.20)		
Yes	3640 (80.07)	1396 (76.37)	1809 (81.82)	435 (85.80)		
Pension insurance, n (%)					χ^2^ = 11.82	**0.003**
No	3169 (69.71)	1225 (67.01)	1592 (72.00)	352 (69.43)		
Yes	1377 (30.29)	603 (32.99)	619 (28.00)	155 (30.57)		
Medical insurance, n (%)					χ^2^ = 2.62	0.269
No	208 (4.58)	91 (4.98)	90 (4.07)	27 (5.33)		
Yes	4338 (95.42)	1737 (95.02)	2121 (95.93)	480 (94.67)		
Social participation, n (%)					χ^2^ = 3.73	0.155
No	2220 (48.83)	865 (47.32)	1112 (50.29)	243 (47.93)		
Yes	2326 (51.17)	963 (52.68)	1099 (49.71)	264 (52.07)		
Smoking status, n (%)					χ^2^ = 28.74	**<0.001**
Never smoker	2700 (59.39)	1172 (64.11)	1247 (56.40)	281 (55.42)		
Former smoker	376 (8.27)	131 (7.17)	196 (8.86)	49 (9.66)		
Current smoker	1470 (32.34)	525 (28.72)	768 (34.74)	177 (34.91)		
Drinking status, n (%)					χ^2^ = 20.27	**<0.001**
Never drinker	2578 (56.71)	1101 (60.23)	1216 (55.00)	261 (51.48)		
Former drinker	287 (6.31)	101 (5.53)	142 (6.42)	44 (8.68)		
Current drinker	1681 (36.98)	626 (34.25)	853 (38.58)	202 (39.84)		

FD: food deprivation, ^#^Kruskal–Waill test, χ^2^: Chi-square test. M: median, Q_1_: 1st Quartile, Q_3_: 3st Quartile. Bold values indicate statistical significance (*P* < 0.05).

### Association between childhood food deprivation and PPC-MM

**FIGURE 2:**
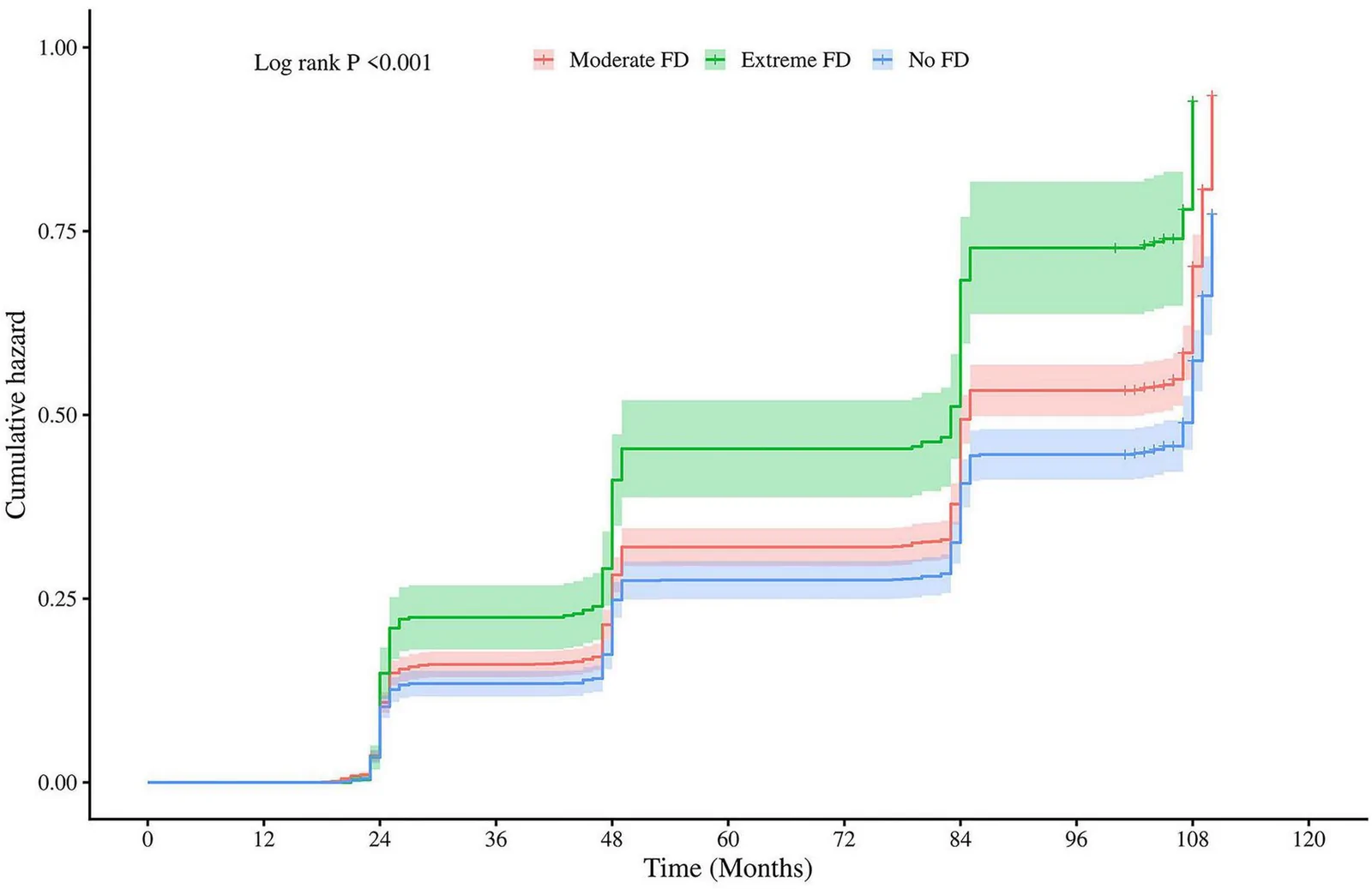
Kaplan–Meier curves for cumulative incidence of physical-psychological-cognitive multimorbidity by childhood food deprivation status.

The relationship between childhood food deprivation and PPC-MM is detailed in Table 2. In the unadjusted model, individuals experiencing moderate childhood food deprivation exhibited an HR of 1.20 (95% CI, 1.10–1.31; *P* < 0.001) for PPC-MM, in comparison to those without such deprivation. Participants with extreme deprivation demonstrated an HR of 1.60 (95% CI, 1.40–1.82; *P* < 0.001). Upon adjustment for demographic variables, the HRs were 1.19 (95% CI, 1.08–1.30) and 1.44 (95% CI, 1.26–1.65), respectively, with both remaining statistically significant (*P* < 0.001). Further adjustments for lifestyle and health-related covariates resulted in HRs of 1.17 (95% CI, 1.07–1.28) and 1.43 (95% CI, 1.25–1.63), respectively, both maintaining significance (*P* < 0.001). Kaplan-Meier survival analysis revealed significant differences in the cumulative incidence of PPC-MM across the three groups (Log-rank test, *P* < 0.001), as illustrated in Figure 2.

**TABLE 2:** Cox regression analysis of childhood food deprivation and physical-psychological-cognitive multimorbidity outcomes.

Variables	Model1		Model2		Model3	
	HR (95%CI)	*P*	HR (95%CI)	*P*	HR (95%CI)	*P*
Food deprivation status						
No FD	1.00 (Reference)		1.00 (Reference)		1.00 (Reference)	
Moderate FD	1.20 (1.10 ∼ 1.31)	**<0.001**	1.19 (1.08 ∼ 1.30)	**<0.001**	1.17 (1.07 ∼ 1.28)	**<0.001**
Extreme FD	1.60 (1.40 ∼ 1.82)	**<0.001**	1.44 (1.26 ∼ 1.65)	**<0.001**	1.43 (1.25 ∼ 1.63)	**<0.001**

HR, hazard ratio, CI, confidence interval, FD, food deprivation. Model1: Crude. Model2: Adjusted for: age group, sex, residence, ethnicity, education level, marital status. Model3: Adjusted for: age group, sex, residence, ethnicity, education level, marital status, maternal agricultural work before age 17, paternal agricultural work before age 17, pension insurance, medical insurance, social participation, smoking status, drinking status, BMI. Bold values indicate statistical significance (*P* < 0.05).

### Sensitivity analyses

To evaluate the robustness of our primary findings, we performed a series of sensitivity analyses. Initially, utilizing the original non-imputed dataset, we found that the association between childhood food deprivation and the incidence of PPC-MM remained consistent with the results of the main analysis. Specifically, both moderate deprivation (HR = 1.17, 95% CI: 1.04–1.31; *P* = 0.009) and extreme deprivation (HR = 1.45, 95% CI: 1.23–1.71; *P* < 0.001) were significantly associated with an increased risk of PPC-MM (see Supplementary Table 4). Subsequently, among participants who were free of any PPC-MM components at baseline, extreme deprivation was significantly correlated with the onset of PPC-MM (HR = 1.50, 95% CI: 1.14–1.97; *P* = 0.004), whereas moderate deprivation did not show a significant association (HR = 1.19, 95% CI: 0.99–1.42; *P* = 0.065) (refer to Supplementary Table 5). In participants presenting with a single baseline disease, extreme deprivation remained significant (HR = 1.27, 95% CI: 1.09–1.48; *P* = 0.002), while moderate deprivation did not reach statistical significance (HR = 1.10, 95% CI: 0.99–1.22; *P* = 0.084) (refer to Supplementary Table 6). Additionally, to address potential circularity between extreme deprivation and famine birth cohort, we excluded the extreme deprivation category and repeated the analysis. The association remained consistent (moderate deprivation: HR = 1.18, 95% CI: 1.08–1.30; *P* < 0.001; refer to Supplementary Table 7).

### Subgroup analyses and the interaction test

Exploratory subgroup analyses were performed, stratified by sex, residence, BMI and age group, to investigate potential effect modification (refer to Table 3). In the context of moderate childhood food deprivation, significant associations with PPC-MM were identified among males (*P* < 0.001), rural residents (*P* = 0.007), urban residents (*P* = 0.027), BMI ≤ 23.9kg/m^2^ (*P* < 0.001) and individuals aged 45–59 years (*P* < 0.001). Conversely, no significant associations were detected among females (*P* = 0.230), BMI > 24.0kg/m^2^ (*P* = 0.225) and individuals aged ≥60 years (*P* = 0.299). Regarding extreme childhood food deprivation, significant associations were found in females (*P* < 0.001), males (*P* < 0.001), rural residents (*P* = 0.004), urban residents (*P* < 0.001), BMI ≤ 23.9kg/m^2^ (*P* < 0.001), BMI > 24.0kg/m^2^ (*P* = 0.002), the 45–59 years age group (*P* < 0.001), and the ≥60 years age group (*P* = 0.024). No significant interaction effects were observed for any stratification variable, as all *P*-values for interaction exceeded 0.05.

**TABLE 3:** Subgroup analysis of the association between childhood food deprivation and physical-psychological-cognitive multimorbidity.

Subgroup	No FD	Moderate FD	Extreme FD	HR (95%CI)^2^	HR (95%CI)^3^	*P* ^2^	*P* ^3^	*P* for interaction
All	803/1828	1122/2211	311/507	1.17 (1.07 ∼ 1.28)	1.43 (1.25 ∼ 1.63)	**<0.001**	**<0.001**	
Sex								0.070
Female	521/986	594/1014	158/220	1.08 (0.96 ∼ 1.21)	1.39 (1.16 ∼ 1.66)	0.230	**<0.001**	
Male	282/842	528/1197	153/287	1.35 (1.17 ∼ 1.57)	1.54 (1.26 ∼ 1.88)	**<0.001**	**<0.001**	
Residence								0.455
Urban	243/727	370/834	81/165	1.25 (1.06 ∼ 1.48)	1.46 (1.13 ∼ 1.88)	**0.007**	**0.004**	
Rural	560/1101	752/1377	230/342	1.13 (1.01 ∼ 1.27)	1.42 (1.22 ∼ 1.66)	**0.027**	**<0.001**	
BMI								0.220
≤23.9	413/977	610/1192	174/283	1.25 (1.10 ∼ 1.42)	1.49 (1.24 ∼ 1.78)	**<0.001**	**<0.001**	
>24.0	390/851	512/1019	137/224	1.09 (0.95 ∼ 1.24)	1.36 (1.11 ∼ 1.66)	0.225	**0.002**	
Age group								0.175
45–59	464/1150	740/1497	163/260	1.24 (1.10 ∼ 1.40)	1.60 (1.34 ∼ 1.92)	**<0.001**	**<0.001**	
≥60	339/678	382/714	148/247	1.08 (0.93 ∼ 1.25)	1.25 (1.03 ∼ 1.53)	0.299	**0.024**	

FD, food deprivation, HR, Hazard Ratio, CI, confidence interval.

^2^ *P* value and HR for moderate food deprivation compared with no food deprivation (reference group).

^3^ *P* value and HR for extreme food deprivation compared with no food deprivation (reference group). Adjusted for: age group, sex, residence, ethnicity, education level, marital status, maternal agricultural work before age 17, paternal agricultural work before age 17, pension insurance, medical insurance, social participation, smoking status, drinking status, BMI. Bold values indicate statistical significance (*P* < 0.05).

### Mediating effect of Light-BioAge on the relationship between childhood food deprivation and PPC-MM

Table 4 presents the results of a causal mediation analysis examining the relationship between childhood food deprivation and the subsequent development of PPC-MM. Both moderate and extreme levels of childhood food deprivation were found to be significantly associated with an increased risk of PPC-MM when compared to the absence of deprivation. Specifically, for moderate deprivation, the total effect was characterized by an HR of 1.20 (95%CI: 1.12–1.28, *P* < 0.001). The direct effect accounted for 95.1% of this total effect, with an HR of 1.19 (95% CI: 1.12–1.27, *P* < 0.001). The indirect effect, mediated through Light-BioAge, had an HR of 1.01 (95% CI: 1.00–1.01, *P* = 0.004), explaining 4.9% of the total effect (95% CI: 1.3%–8.5%). In the case of extreme deprivation, the total effect was an HR of 1.45 (95% CI: 1.28–1.64, *P* < 0.001), with the direct effect contributing 94.7% of the total effect (HR 1.43, 95% CI: 1.26–1.62, *P* < 0.001). The indirect effect was characterized by an HR of 1.02 (95% CI: 1.01–1.03, *P* = 0.004), accounting for 5.3% of the total effect (95% CI: 1.5%–9.2%). The equivalence of the controlled direct effect and the natural direct effect in both scenarios supports the assumption of no interaction between the exposure and the mediator.

**TABLE 4:** Causal mediation analysis of Light-BioAge in the association between childhood food deprivation and physical-psychological-cognitive multimorbidity.

Estimated effect	Moderate FD vs. No FD		Extreme FD vs. No FD	
	HR (95% CI)	*P* value	HR (95% CI)	*P* value
Total effect	1.20 (1.12∼1.28)	**<0.001**	1.45 (1.28∼1.64)	**<0.001**
Direct effect	1.19 (1.12∼1.27)	**<0.001**	1.43 (1.26∼1.62)	**<0.001**
Indirect effect	1.01 (1.00∼1.01)	**0.004**	1.02 (1.01∼1.03)	**0.004**
Proportion mediated, % (95% CI)	4.9 (1.3∼8.5)	–	5.3 (1.5∼9.2)	–

FD, food deprivation, HR, hazard ratio; CI, confidence interval. Adjusted for: age group, sex, residence, ethnicity, education level, marital status, maternal agricultural work before age 17, paternal agricultural work before age 17, pension insurance, medical insurance, social participation, smoking status, drinking status, BMI. Bold values indicate statistical significance (*P* < 0.05).

We further conducted a sensitivity mediation analysis using Light-BioAge minus chronological age as an age-independent mediator. For moderate deprivation, the total effect was an HR of 1.20 (95% CI: 1.13–1.28, *P* < 0.001). Of this, the direct effect was 1.20 (95% CI: 1.13–1.28, *P* < 0.001) and the indirect effect was 1.00 (95% CI: 1.00–1.01, *P* = 0.174), accounting for 1.60% of the total effect. For extreme deprivation, the total effect was 1.45 (95% CI: 1.27–1.64, *P* < 0.001), the direct effect was 1.44 (95% CI: 1.27–1.63, *P* < 0.001), and the indirect effect was 1.01 (95% CI: 1.00–1.01, *P* = 0.174), accounting for 1.74% of the total effect. Detailed results are presented in Supplementary Table 11.

## Discussion

The study found that childhood food deprivation was significantly associated with the risk of PPC-MM, showing a dose-response relationship, with the highest risk observed among individuals with extreme deprivation (HR = 1.43) and an increased risk among those with moderate deprivation (HR = 1.17). This finding is highly consistent with the Developmental Origins of Health and Disease (DOHaD) theoretical framework, which posits that early-life environmental exposures leave persistent biological imprints through mechanisms such as epigenetic modification (30), metabolic programming (31), and developmental plasticity of organ systems (32), thereby increasing susceptibility to various chronic diseases in adulthood (33, 34). Cohort study evidence indicates that famine or early nutritional deprivation exerts lasting harm on long-term health, with childhood representing a critical susceptible window. The Dutch Famine Study showed that individuals exposed at ages 11–14 had higher risks of diabetes and/or peripheral arterial disease at ages 60-76 (35); the Leningrad Siege Study found that childhood and adolescent exposure was associated with elevated systolic blood pressure and circulatory diseases in the long term (36). Additionally, childhood food deprivation exposure has been linked to increased risk of cognitive impairment (37), and individuals who experienced childhood hunger showed significantly higher frailty risk in middle-aged and older adulthood (16). However, these studies have largely focused on single health outcomes. This study is the first to systematically examine childhood food deprivation within the three-dimensional framework of PPC-MM. Utilizing longitudinal follow-up data from the CHARLS cohort from 2011 to 2020, with childhood food deprivation as the baseline exposure and incident PPC-MM during follow-up as the outcome, we may have helped mitigate reverse causation bias and provided a more rigorous evidence base for examining the longitudinal association between early nutritional exposure and late-life composite health outcomes. Furthermore, by simultaneously adjusting for childhood socioeconomic indicators (parental occupation) and adult socioeconomic indicators (education, insurance, residence), this study supports the interpretation that the effect of childhood food deprivation may be partially independent of subsequent social mobility.

Subgroup analyses revealed that the association between childhood food deprivation and PPC-MM was directionally consistent across strata of adult characteristics, but the effect size varied, with no significant interaction observed for any stratification variable (all P for interaction > 0.05). These subgroup findings are exploratory. Moderate deprivation was associated with PPC-MM in males (HR = 1.35, 95% CI: 1.17–1.57) but not in females (HR = 1.08, 95% CI: 0.96–1.21), with a non-significant interaction (*P* = 0.070). This sex difference may reflect distinct stress responses: males may show stronger metabolic adaptation to early adversity, while estrogen may protect females from long-term nutritional deprivation effects. However, this interpretation is speculative, given the non-significant interaction and the significant effect of severe deprivation in both sexes. Moderate deprivation was significant in the 45–59 years group (*P* < 0.001) but not in the ≥60 years group (*P* = 0.299), although the overall interaction was non-significant (*P* = 0.175). This observation may suggest modification by developmental sensitive period, as some members of the 45–59 years group were in fetal or early childhood during the Great Famine, a critical window for metabolic programming. Previous studies (38–40) and a recent meta-analysis (41) reported that fetal exposure to famine increased the risk of adult metabolic diseases, supporting the fetal programming hypothesis (34). Extreme deprivation was significant in both age groups due to its stringent definition ensuring consistent exposure intensity. The BMI interaction was non-significant (*P* = 0.220). Both moderate and severe deprivation were significant in the normal/underweight group. Among overweight/obese participants, only severe deprivation was significant (*P* = 0.002), with moderate deprivation showing no association (*P* = 0.225). Lu et al. (39) reported that the association between fetal famine exposure and diabetes was amplified in individuals with BMI ≥ 24 kg/m^2^, but the interaction in this study was non-significant (*P* = 0.220), suggesting that overweight/obesity itself as a strong risk factor may mask the independent effect of moderate deprivation, with only extreme exposure able to break through the metabolic reserve threshold. Residential stratification showed significant associations in both rural and urban areas, with no significant interaction (*P* = 0.455). The above subgroup analyses are all exploratory findings awaiting validation.

This study exploratorily examined the potential mediating role of Light-BioAge in the association between childhood food deprivation and PPC-MM. In the main analysis, the direct effect accounted for 94.7%–95.1% of the total effect, while the indirect effect through Light-BioAge explained only 4.9%–5.3%. However, in sensitivity analyses using Light-BioAge minus chronological age as an age-independent mediator, the indirect effects became non-significant (HR = 1.00–1.01, *P* = 0.174), with the proportion mediated dropping to 1.60%–1.74%. This suggests that the apparent mediation effect observed in the main analysis may largely reflect the mechanical contribution of chronological age embedded in the Light-BioAge algorithm, rather than a true biological aging mechanism. This finding is consistent with prior literature showing limited or null mediating effects of biological age indicators in adversity-health associations. For example, Klopack et al. (42) reported that GrimAge explained 9%–14% of the association between ACEs and depressive symptoms, DunedinPoAm38 explained 2%–7%, while PhenoAge showed no significant mediating effect; Martinez et al. (43) similarly found no significant mediation by biological age in the association between childhood adversity and depression. The smaller effect size observed in our study may reflect the simplified nature of Light-BioAge, which integrates only three clinical parameters (creatinine, glucose, and C-reactive protein) and may not fully capture the multidimensional aging processes relevant to multimorbidity development. Moreover, the age-independent sensitivity analysis suggests that even this small apparent effect may largely reflect the mechanical contribution of chronological age. Furthermore, single baseline measurement cannot reflect dynamic aging trajectories, potentially underestimating cumulative mediating effects. The non-significant mediating effect of biological age also suggests that other pathways may play more important roles. Klopack et al. (42) found that socioeconomic factors and lifestyle (obesity, substance use) could attenuate the mediating effect of epigenetic aging, indicating the regulatory role of behavioral pathways; while cognitive impairment (14), physical frailty (16), and metabolic programming changes (30, 31) may also independently or synergistically contribute to the three-dimensional damage pattern of PPC-MM. This study employed causal mediation analysis within the Cox framework, which better handles temporal dynamics than traditional methods, but limitations including observational design, cross-sectional measurement, the intrinsic dependence of Light-BioAge on chronological age, and limited clinical significance of the small mediation proportion should be acknowledged. Future research should employ biological age indicators independent of chronological age, repeatedly measure biological age across multiple follow-up waves and explore other potential mediating factors.

This study possesses several notable strengths. Firstly, it employed a national prospective cohort (CHARLS) characterized by a large sample size and an extended follow-up period, thereby enhancing its generalizability. Secondly, it utilized a three-dimensional PPC-MM composite outcome, which more accurately represents the complexity of multimorbidity in older adults. Thirdly, the study introduced the innovative concept of Light-BioAge and applied mediation analysis within the Cox framework, offering a methodological perspective. Nonetheless, several limitations must be acknowledged. Firstly, childhood food deprivation was retrospectively self-reported over 30–70 years, subject to recall bias and survivorship bias; the Great Famine caused substantial excess mortality, and our sample includes only survivors who lived to age 45 years or older, which may have attenuated the true association. Secondly, we did not account for competing risks from mortality; participants who died before developing PPC-MM were treated as censored, potentially underestimating the association. Future studies should prioritize competing risk analyses to obtain more accurate cumulative incidence estimates. Thirdly, baseline covariates measured in 2011, decades after the childhood exposure, may not fully satisfy the classical confounder definition. Fourthly, the validity of mediation may be constrained by the single-timepoint measurement of Light-BioAge, the intrinsic dependence of Light-BioAge on chronological age and the presence of unmeasured confounding variables, thus this finding should be considered exploratory. Fifthly, the observational design precludes strict causal inferences.

## Conclusion

In conclusion, this study, utilizing data from the CHARLS database, establishes a robust longitudinal dose-response association between childhood food deprivation and PPC-MM within a nationally representative Chinese cohort. The observed association follows a dose-response pattern, with extreme deprivation posing the highest risk, and remains robust after adjusting for multiple covariates. Subgroup analyses indicated a consistent direction of association across various adult characteristics, although effect sizes differed by sex, age, and BMI categories. Light-BioAge showed a small, exploratory mediating effect with indirect effects close to the null, suggesting that other pathways likely play more important roles. These findings underscore the importance of incorporating early-life risk assessment into strategies for promoting healthy aging. Future research should focus on employing multidimensional aging indicators, conducting repeated longitudinal assessments, and undertaking intervention studies to further elucidate underlying mechanisms and facilitate the translation of these findings into public health practice.

## Data Availability

Publicly available datasets were analyzed in this study. This data can be found here: http://charls.pku.edu.cn.

## References

[1] Kumar A Yadav S. Towards a new perspective: exploring the variability of conditional risk factors for multimorbidity susceptibility among older adults in India. *PLoS One*. (2025) 20:e0323890. 10.1371/journal.pone.0323890 40526621 PMC12173406

[2] Tan MMC Hanlon C Muniz-Terrera G Benaglia T Ismail R Mohan D et al. Multimorbidity latent classes in relation to 11-year mortality, risk factors and health-related quality of life in Malaysia: a prospective health and demographic surveillance system study. *BMC Med*. (2025) 23:5. 10.1186/s12916-024-03796-z 39757194 PMC11702131

[3] Han S Chen W Shen M Shao R Yang W Wang C. Multimorbidity progression and the heterogeneous impact of healthy ageing risk factors: a multicohort study. *BMJ Public Health*. (2025) 3:e002474. 10.1136/bmjph-2024-002474 40761358 PMC12320038

[4] Yang L Zhang Z Zhang J Miao J Zhang H Du Y et al. Stage-specific risk factors of cardiometabolic multimorbidity: a systematic review and meta-analysis from incidence to mortality. *Ageing Res Rev*. (2026) 114:102991. 10.1016/j.arr.2025.102991 41390099

[5] Lin L Duan DF Yan L He HY. Prevalence and associated factors of physical-psychological-cognitive multimorbidity in Chinese community-dwelling older adults: a cross-sectional study. *PeerJ*. (2025) 13:e19750. 10.7717/peerj.19750 40718771 PMC12296576

[6] Pan Y Bi J Sun L Feng L Wang R Guo Z et al. Subjective well-being and allostatic load in multimorbidity transitions: a multi-state survival analysis of three international longitudinal cohorts. *Gen Hosp Psychiatry*. (2026) 99:171–8. 10.1016/j.genhosppsych.2026.02.003 41678898

[7] Bi J Pan Y Guo W Ji P Xing Z Feng L et al. Association of circadian syndrome with the risk of physical, psychological, and cognitive multimorbidities: a prospective cohort study based on the China health and retirement longitudinal study. *J Glob Health*. (2025) 15:04351. 10.7189/jogh.15.04351 41411529 PMC12714357

[8] Pavela G Latham K. Childhood conditions and multimorbidity among older adults. *J Gerontol B Psychol Sci Soc Sci*. (2016) 71:889–901. 10.1093/geronb/gbv028 25975290 PMC4982384

[9] Umberson D Williams K Thomas PA Liu H Thomeer MB. Race, gender, and chains of disadvantage: childhood adversity, social relationships, and health. *J Health Soc Behav*. (2014) 55:20–38. 10.1177/0022146514521426 24578394 PMC4193500

[10] Meng P. Cumulative childhood adversity and frailty in middle and old age among socioeconomic groups in China: a retrospective study. *Geriatr Nurs*. (2025) 65:103459. 10.1016/j.gerinurse.2025.103459 40616928

[11] Dai Q Li M Wang Z Xu Q Zhang X Tao L. The mediating effects of chronic diseases in the relationship between adverse childhood experiences and trajectories of depressive symptoms in later life: a nationwide longitudinal study. *Healthcare*. (2024) 12:2539. 10.3390/healthcare12242539 39765965 PMC11675985

[12] Xu J Han G Xu X. Adverse childhood experiences and 10-year depressive-symptoms trajectories among middle-aged and older adults in China: a population-based cohort study. *Front Public Health*. (2024) 12:1455750. 10.3389/fpubh.2024.1455750 39717034 PMC11663716

[13] Zhou X Liu X Tan Y Wang J. Does social participation buffer the association? Adverse childhood experiences and 10-year depression symptom trajectories in middle-aged and older Chinese. *J Affect Disord*. (2026) 397:120897. 10.1016/j.jad.2025.120897 41422958

[14] Cohn-Schwartz E Weinstein G. Early-life food deprivation and cognitive performance among older Europeans. *Maturitas*. (2020) 141:26–32. 10.1016/j.maturitas.2020.06.020 33036699

[15] Wang XC Huang ZP. Adverse childhood experiences and subsequent impact on adulthood cognitive impairment: a systematic review and meta-analysis. *Front Psychiatry*. (2026) 16:1751619. 10.3389/fpsyt.2025.1751619 41647757 PMC12869310

[16] Ye C Aihemaitijiang S Wang R Halimulati M Zhang Z. Associations between early-life food deprivation and risk of frailty of middle-age and elderly people: evidence from the China health and retirement longitudinal study. *Nutrients*. (2021) 13:3066. 10.3390/nu13093066 34578943 PMC8472025

[17] Cui H Smith JP Zhao Y. Early-life deprivation and health outcomes in adulthood: evidence from childhood hunger episodes of middle-aged and elderly Chinese. *J Dev Econ*. (2020) 143:102417. 10.1016/j.jdeveco.2019.102417 32863534 PMC7451260

[18] Li D Guo X Zhang W Li W Zhang T Liu Z et al. The association between childhood hunger experiences and health in middle and old age: a longitudinal study over 10 years. *BMC Public Health*. (2025) 25:193. 10.1186/s12889-025-21345-y 39819302 PMC11740438

[19] Shalev I Belsky J. Early-life stress and reproductive cost: a two-hit developmental model of accelerated aging? *Med Hypotheses*. (2016) 90:41–7. 10.1016/j.mehy.2016.03.002 27063083

[20] Engdahl E Alavian-Ghavanini A Forsell Y Lavebratt C Rüegg J. Childhood adversity increases methylation in the GRIN2B gene. *J Psychiatr Res*. (2021) 132:38–43. 10.1016/j.jpsychires.2020.09.022 33038564

[21] Battista JT Piacentino D Schwandt ML Lee MR Faulkner ML Farokhnia M et al. Investigating the relationship between early life adversity, inflammation and alcohol use. *Addict Biol*. (2023) 28:e13274. 10.1111/adb.13274 37186442 PMC10214493

[22] Hao M Zhang H Wu J Huang Y Li X Wang M et al. Gompertz law-based biological age (GOLD BioAge): a simple practical measurement of biological ageing to capture morbidity and mortality risks. *Adv Sci*. (2025) 12:e01765. 10.1002/advs.202501765 40600465 PMC12407260

[23] Zhao YS John Y Gonghuan G John H Peifeng H Yisong L et al. *China Health and Retirement Longitudinal Study: 2011-2012 National Baseline User’s Guide.* Beijing: National School of Development, Peking University (2013).

[24] Zhou Y Kivimäki M Holt-Lunstad J Yan LL Zhang Y Wang H et al. Stressful life events in childhood and adulthood and risk of physical, psychological and cognitive multimorbidities: a multicohort study. *EClinicalMedicine*. (2025) 83:103225. 10.1016/j.eclinm.2025.103225 40453536 PMC12123342

[25] Ni Y Zhou Y Kivimäki M Cai Y Carrillo-Larco RM Xu X et al. Socioeconomic inequalities in physical, psychological, and cognitive multimorbidity in middle-aged and older adults in 33 countries: a cross-sectional study. *Lancet Healthy Longev*. (2023) 4:e618–28. (23)00195-2 10.1016/S2666-7568(23)00195-2 37924843

[26] Zhou Y Kivimäki M Yan LL Carrillo-Larco RM Zhang Y Cheng Y et al. Associations between socioeconomic inequalities and progression to psychological and cognitive multimorbidities after onset of a physical condition: a multicohort study. *EClinicalMedicine*. (2024) 74:102739. 10.1016/j.eclinm.2024.102739 39157288 PMC11327438

[27] Chen J Zhou W Huang Y. Association between serum uric acid levels and depressive symptoms according to menopausal status. *J Affect Disord*. (2024) 350:240–6. 10.1016/j.jad.2024.01.108 38220113

[28] Meng Q Wang H Strauss J Langa KM Chen X Wang M et al. Validation of neuropsychological tests for the China health and retirement longitudinal study harmonized cognitive assessment protocol. *Int Psychogeriatr*. (2019) 31:1709–19. 10.1017/S1041610219000693 31309907 PMC8082093

[29] Liu W Fan L Shi D Yu L Song J Liang R et al. Physiological measures variability and risks of heart disease and stroke: evidence from three cohort studies. *BMC Med*. (2024) 22:590. 10.1186/s12916-024-03805-1 39695684 PMC11657114

[30] Godfrey KM Lillycrop KA Burdge GC Gluckman PD Hanson MA. Epigenetic mechanisms and the mismatch concept of the developmental origins of health and disease. *Pediatr Res.* (2007) 61:5R–10R. 10.1203/pdr.0b013e318045bedb 17413851

[31] Barker DJ. Fetal programming of coronary heart disease. *Trends Endocrinol Metab*. (2002) 13:364–8. (02)00689-6 10.1016/s1043-2760(02)00689-6 12367816

[32] Bateson P Barker D Clutton-Brock T Deb D D’Udine B Foley RA et al. Developmental plasticity and human health. *Nature*. (2004) 430:419–21. 10.1038/nature02725 15269759

[33] Gluckman PD Hanson MA Bateson P Beedle AS Law CM Bhutta ZA et al. Towards a new developmental synthesis: adaptive developmental plasticity and human disease. *Lancet*. (2009) 373:1654–7. (09)60234-8 10.1016/S0140-6736(09)60234-8 19427960

[34] Hales CN Barker DJ. The thrifty phenotype hypothesis. *Br Med Bull*. (2001) 60:5–20. 10.1093/bmb/60.1.5 11809615

[35] Portrait F Teeuwiszen E Deeg D. Early life undernutrition and chronic diseases at older ages: the effects of the dutch famine on cardiovascular diseases and diabetes. *Soc Sci Med*. (2011) 73:711–8. 10.1016/j.socscimed.2011.04.005 21816529

[36] Koupil I Shestov DB Sparén P Plavinskaja S Parfenova N Vågerö D. Blood pressure, hypertension and mortality from circulatory disease in men and women who survived the siege of leningrad. *Eur J Epidemiol*. (2007) 22:223–34. 10.1007/s10654-007-9113-6 17436055

[37] Wang XJ Xu W Li JQ Cao XP Tan L Yu JT. Early-life risk factors for dementia and cognitive impairment in later life: a systematic review and meta-analysis. *J Alzheimers Dis*. (2019) 67:221–9. 10.3233/JAD-180856 30636739

[38] Jiang H Yu Y Li L Xu W. Exposure to the great famine in early life and the risk of obesity in adulthood: a report based on the China health and nutrition survey. *Nutrients*. (2021) 13:1285. 10.3390/nu13041285 33919739 PMC8070734

[39] Lu J Li M Xu Y Bi Y Qin Y Li Q et al. Early life famine exposure, ideal cardiovascular health metrics, and risk of incident diabetes: findings from the 4c study. *Diabetes Care*. (2020) 43:1902–9. 10.2337/dc19-2325 32499384

[40] Zhang W Luan R. Early-life exposure to the Chinese famine of 1959-61 and risk of Hyperuricemia: results from the China health and retirement longitudinal study. *BMC Public Health*. (2020) 20:15. 10.1186/s12889-019-8017-1 31906901 PMC6945412

[41] Hidayat K Du X Shi BM Qin LQ. Foetal and childhood exposure to famine and the risks of cardiometabolic conditions in adulthood: a systematic review and meta-analysis of observational studies. *Obes Rev*. (2020) 21:e12981. 10.1111/obr.12981 32048436

[42] Klopack ET Crimmins EM Cole SW Seeman TE Carroll JE. Accelerated epigenetic aging mediates link between adverse childhood experiences and depressive symptoms in older adults: results from the health and retirement study. *SSM Popul Health*. (2022) 17:101071. 10.1016/j.ssmph.2022.101071 35313610 PMC8933834

[43] Martinez RAM Howard AG Fernández-Rhodes L Maselko J Pence BW Dhingra R et al. Does biological age mediate the relationship between childhood adversity and depression? Insights from the detroit neighborhood health study. *Soc Sci Med*. (2024) 340:116440. 10.1016/j.socscimed.2023.116440 38039767 PMC10843850

