# Supplementary material for: Early-life nutritional deprivation and multimorbidity in middle- aged and older adults: a longitudinal exploratory mediation analysis of biological aging
Source: Front Nutr. 2026 Sep 10;13:1908542. doi: 10.3389/fnut.2026.1908542 (PMC13600986; doi:10.3389/fnut.2026.1908542)
Supplement: Supplementary file 1 [file Table_1.docx]

TableS1 Missing Data Patterns for Covariates Before Imputation.

| **Variables** | **Total** | **Freq** | **Miss (Percent)** |
| --- | --- | --- | --- |
| BMI | 4,546 | 4,043 | 503 (11.06) |
| Maternal agricultural work before age 17 | 4,546 | 4,047 | 499 (10.98) |
| Paternal agricultural work before age 17 | 4,546 | 4,294 | 252 (5.54) |
| Pension insurance | 4,546 | 3,825 | 721 (15.86) |
| Medical insurance | 4,546 | 4,539 | 7 (0.15) |
| Social participation | 4,546 | 4,545 | 1 (0.02) |
| Drinking status | 4,546 | 4,545 | 1 (0.02) |

TableS2 Variance Inflation Factors (VIFs) for the Independent Variables.

|  | **GVIF** | **Df** | **GVIF1/2Df** |
| --- | --- | --- | --- |
| Childhood Food Deprivation | 1.051 | 2 | 1.013 |
| Marital status | 1.051 | 1 | 1.025 |
| Maternal agricultural work before age 17 | 1.424 | 1 | 1.193 |
| Paternal agricultural work before age 17 | 1.438 | 1 | 1.199 |
| Pension insurance | 1.324 | 1 | 1.151 |
| Medical insurance | 1.008 | 1 | 1.004 |
| Social participation | 1.037 | 1 | 1.018 |
| Gender | 2.382 | 1 | 1.543 |
| Ethnicity | 1.008 | 1 | 1.004 |
| Residenc | 1.198 | 1 | 1.094 |
| Education level | 1.34 | 3 | 1.05 |
| Smoking status | 2.002 | 2 | 1.19 |
| Drinking status | 1.488 | 2 | 1.104 |
| Age group | 1.319 | 1 | 1.149 |
| BMI | 1.081 | 1 | 1.039 |

TableS3 Equal-ratio risk assumption test

|  | **Chisg** | **Df** | ***P*** |
| --- | --- | --- | --- |
| Childhood Food Deprivation | 0.523 | 2 | 0.77 |
| Marital status | 0.045 | 1 | 0.833 |
| Maternal agricultural work before age 17 | 1.615 | 1 | 0.204 |
| Paternal agricultural work before age 17 | 1.733 | 1 | 0.188 |
| Pension insurance | 0.078 | 1 | 0.779 |
| Medical insurance | 0.228 | 1 | 0.633 |
| Social participation | 3.568 | 1 | 0.059 |
| Gender | 0.347 | 1 | 0.556 |
| Ethnicity | 1.036 | 1 | 0.309 |
| Residenc | 0.032 | 1 | 0.857 |
| Education level | 0.075 | 3 | 0.995 |
| Smoking status | 0.213 | 2 | 0.899 |
| Drinking status | 4.081 | 2 | 0.13 |
| Age group | 2.319 | 1 | 0.128 |
| BMI | 0.001 | 1 | 0.976 |
| GLOBAL | 19.260 | 20 | 0.505 |

TableS4 Cox regression analysis of childhood food deprivation and physical-psychological-cognitive multimorbidity outcomes in the original (non-imputed) dataset(n=2868).

| Variables | Model1 | |  | Model2 | |  | Model3 | |
| --- | --- | --- | --- | --- | --- | --- | --- | --- |
|  | HR (95%CI) | *P* |  | HR (95%CI) | *P* |  | HR (95%CI) | *P* |
| Food Deprivation Status |  |  |  |  |  |  |  |  |
| No FD | 1.00 (Reference) |  |  | 1.00 (Reference) |  |  | 1.00 (Reference) |  |
| Moderate FD | 1.20 (1.10 ~ 1.31) | **<0.001** |  | 1.19 (1.08 ~ 1.30) | **<0.001** |  | 1.17 (1.04 ~ 1.31) | **0.009** |
| Extreme FD | 1.60 (1.40 ~ 1.82) | **<0.001** |  | 1.44 (1.26 ~ 1.65) | **<0.001** |  | 1.45 (1.23 ~ 1.71) | **<0.001** |
| HR: Hazard Ratio, CI: Confidence Interval, FD: Food Deprivation. | | | | | | | | |
| Model1: Crude | | | | | | | | |
| Model2: Adjust: age group, gender, residence, ethnicity, education level, marital status. | | | | | | | | |
| Model3: Adjust: age group, gender, residence, ethnicity, education level, marital status, maternal agricultural work before age 17, paternal agricultural work before age 17, pension insurance, medical insurance, social participation, smoking status, drinking status, BMI. | | | | | | | | |

TableS5 Cox regression analysis of childhood food deprivation and physical-psychological-cognitive multimorbidity in the baseline disease-free cohort (n=1753).

| Variables | Model1 | |  | Model2 | |  | Model3 | |
| --- | --- | --- | --- | --- | --- | --- | --- | --- |
|  | HR (95%CI) | *P* |  | HR (95%CI) | *P* |  | HR (95%CI) | *P* |
| Food Deprivation Status |  |  |  |  |  |  |  |  |
| No FD | 1.00 (Reference) |  |  | 1.00 (Reference) |  |  | 1.00 (Reference) |  |
| Moderate FD | 1.24 (1.04 ~ 1.49) | **0.018** |  | 1.19 (0.99 ~ 1.42) | 0.062 |  | 1.19 (0.99 ~ 1.42) | 0.065 |
| Extreme FD | 1.78 (1.36 ~ 2.33) | **<0.001** |  | 1.52 (1.16 ~ 2.00) | **0.003** |  | 1.50 (1.14 ~ 1.97) | **0.004** |
| HR: Hazard Ratio, CI: Confidence Interval, FD: Food Deprivation. | | | | | | | | |
| Model1: Crude | | | | | | | | |
| Model2: Adjust: age group, gender, residence, ethnicity, education level, marital status. | | | | | | | | |
| Model3: Adjust: age group, gender, residence, ethnicity, education level, marital status, maternal agricultural work before age 17, paternal agricultural work before age 17, pension insurance, medical insurance, social participation, smoking status, drinking status, BMI. | | | | | | | | |

TableS6 Cox regression analysis of childhood food deprivation and physical-psychological-cognitive multimorbidity in the single-disease baseline group (n=2793).

| Variables | Model1 | |  | Model2 | |  | Model3 | |
| --- | --- | --- | --- | --- | --- | --- | --- | --- |
|  | HR (95%CI) | *P* |  | HR (95%CI) | *P* |  | HR (95%CI) | *P* |
| Food Deprivation Status |  |  |  |  |  |  |  |  |
| No FD | 1.00 (Reference) |  |  | 1.00 (Reference) |  |  | 1.00 (Reference) |  |
| Moderate FD | 1.11 (1.00 ~ 1.23) | 0.055 |  | 1.11 (1.00 ~ 1.23) | 0.058 |  | 1.10 (0.99 ~ 1.22) | 0.084 |
| Extreme FD | 1.37 (1.18 ~ 1.59) | **< 0.001** |  | 1.28 (1.10 ~ 1.49) | **0.002** |  | 1.27 (1.09 ~ 1.48) | **0.002** |
| HR: Hazard Ratio, CI: Confidence Interval, FD: Food Deprivation.  Model1: Crude  Model2: Adjust: age group, gender, residence, ethnicity, education level, marital status.  Model3: Adjust: age group, gender, residence, ethnicity, education level, marital status, maternal agricultural work before age 17, paternal agricultural work before age 17, pension insurance, medical insurance, social participation, smoking status, drinking status, BMI. | | | | | | | | |

TableS7 Cox regression analysis of childhood food deprivation and physical-psychological-cognitive multimorbidity after excluding the extreme-deprivation group (n=4,036).

| Variables | Model1 | |  | Model2 | |  | Model3 | |
| --- | --- | --- | --- | --- | --- | --- | --- | --- |
|  | HR (95%CI) | *P* |  | HR (95%CI) | *P* |  | HR (95%CI) | *P* |
| Food Deprivation Status |  |  |  |  |  |  |  |  |
| No FD | 1.00 (Reference) |  |  | 1.00 (Reference) |  |  | 1.00 (Reference) |  |
| Moderate FD | 1.20 (1.09 ~ 1.31) | **<0.001** |  | 1.20 (1.09 ~ 1.31) | **<0.001** |  | 1.18 (1.08 ~ 1.30) | **<0.001** |
| HR: Hazard Ratio, CI: Confidence Interval, FD: Food Deprivation.  Model1: Crude  Model2: Adjust: age group, gender, residence, ethnicity, education level, marital status.  Model3: Adjust: age group, gender, residence, ethnicity, education level, marital status, maternal agricultural work before age 17, paternal agricultural work before age 17, pension insurance, medical insurance, social participation, smoking status, drinking status, BMI. | | | | | | | | |

Table S8. Comparison of baseline characteristics between excluded and retained participants.

| Variables | Retained  (n = 6243) | Excluded  (n = 4546) | Statistic | *P* |
| --- | --- | --- | --- | --- |
|  |  |  |  |  |
| BMI (kg/m^2^),  M (Q₁, Q₃) | 23.40 (21.17, 25.78) | 23.63 (21.35, 26.05) | Z=-3.08 | **0.002** |
| Age (year)  M (Q₁, Q₃) | 56.00 (50.00, 62.00) | 57.00 (50.00, 62.00) | Z=-0.60 | 0.547 |
| Sex, n (%) |  |  | χ²=0.86 | 0.355 |
| Female | 3105 (49.74) | 2220 (48.83) |  |  |
| Male | 3138 (50.26) | 2326 (51.17) |  |  |
| Ethnicity, n (%) |  |  | χ²=0.65 | 0.420 |
| Han Chinese | 5851 (93.72) | 4243 (93.33) |  |  |
| Ethnic minorities | 392 (6.28) | 303 (6.67) |  |  |
| Residence, n (%) |  |  | χ²=0.88 | 0.347 |
| Urban | 2426 (38.86) | 1726 (37.97) |  |  |
| Rural | 3817 (61.14) | 2820 (62.03) |  |  |
| Marital status, n(%) |  |  | χ²=0.27 | 0.606 |
| Married | 485 (7.77) | 341 (7.50) |  |  |
| Other marital statuses | 5758 (92.23) | 4205 (92.50) |  |  |
| Education level, n(%) |  |  | χ²=3.39 | 0.335 |
| Illiterate | 2387 (38.23) | 1795 (39.49) |  |  |
| Primary school | 1468 (23.51) | 1071 (23.56) |  |  |
| Middle school | 1497 (23.98) | 1081 (23.78) |  |  |
| High school and above | 891 (14.27) | 599 (13.18) |  |  |
| Maternal agricultural work before age 17, n(%) |  |  | χ²=4.06 | **0.044** |
| No | 490 (7.85) | 310 (6.82) |  |  |
| Yes | 5753 (92.15) | 4236 (93.18) |  |  |
| Paternal agricultural work before age 17, n(%) |  |  | χ²=0.12 | 0.731 |
| No | 1261 (20.20) | 906 (19.93) |  |  |
| Yes | 4982 (79.80) | 3640 (80.07) |  |  |
| Pension insurance, n(%) |  |  | χ²=16.85 | **<0.001** |
| No | 4118 (65.96) | 3169 (69.71) |  |  |
| Yes | 2125 (34.04) | 1377 (30.29) |  |  |
| Medical insurance, n(%) |  |  | χ²=0.88 | 0.349 |
| No | 310 (4.97) | 208 (4.58) |  |  |
| Yes | 5933 (95.03) | 4338 (95.42) |  |  |
| Social participation, n(%) |  |  | χ²=0.86 | 0.355 |
| No | 3105 (49.74) | 2220 (48.83) |  |  |
| Yes | 3138 (50.26) | 2326 (51.17) |  |  |
| Smoking status, n(%) |  |  | χ²=0.48 | 0.788 |
| Never smoker | 3669 (58.77) | 2700 (59.39) |  |  |
| Former smoker | 517 (8.28) | 376 (8.27) |  |  |
| Current smoker | 2057 (32.95) | 1470 (32.34) |  |  |
| Drinking status, n(%) |  |  | χ²=0.16 | 0.922 |
| Never drinker | 3518 (56.35) | 2578 (56.71) |  |  |
| Former drinker | 402 (6.44) | 287 (6.31) |  |  |
| Current drinker | 2323 (37.21) | 1681 (36.98) |  |  |
| Food Deprivation Status |  |  | χ²=0.07 | 0.967 |
| No FD | 2495 (39.96) | 1828 (40.21) |  |  |
| Moderate FD | 3050 (48.85) | 2211 (48.64) |  |  |
| Extreme FD | 698 (11.18) | 507 (11.15) |  |  |
| Z: Mann-Whitney test, χ²: Chi-square test | | | | |
| M: Median, Q₁: 1st Quartile, Q₃: 3st Quartile | | | | |

Table S9. Cox Regression Analysis of Childhood Food Deprivation and Physical-Psychological-Cognitive Multimorbidity Outcomes (n= 6243).

| Variables | Model1 | |  | Model2 | |  | Model3 | |
| --- | --- | --- | --- | --- | --- | --- | --- | --- |
|  | HR (95%CI) | *P* |  | HR (95%CI) | *P* |  | HR (95%CI) | *P* |
| Food Deprivation Status |  |  |  |  |  |  |  |  |
| No FD | 1.00 (Reference) |  |  | 1.00 (Reference) |  |  | 1.00 (Reference) |  |
| Moderate FD | 1.19 (1.10 ~ 1.28) | **<0.001** |  | 1.16 (1.07 ~ 1.25) | **<0.001** |  | 1.15 (1.06 ~ 1.24) | **<0.001** |
| Extreme FD | 1.50 (1.34 ~ 1.68) | **<0.001** |  | 1.35 (1.20 ~ 1.51) | **<0.001** |  | 1.33 (1.19 ~ 1.50) | **<0.001** |
| HR: Hazard Ratio, CI: Confidence Interval, FD: Food Deprivation.  Model1: Crude.  Model2: Adjusted for: age group, sex, residence, ethnicity, education level, marital status.  Model3: Adjusted for: age group, sex, residence, ethnicity, education level, marital status, maternal agricultural work before age 17, paternal agricultural work before age 17, pension insurance, medical insurance, social participation, smoking status, drinking status, BMI. | | | | | | | | |

Table S10. Landmark causal mediation analysis of Light-BioAge in the association between childhood food deprivation and incident physical-psychological-cognitive multimorbidity (2015–2020 follow-up, n=2,806).

| Estimated effect | Moderate FD vs No FD | | Extreme FD vs No FD | |
| --- | --- | --- | --- | --- |
|  | HR (95% CI) | *P* value | HR (95% CI) | *P* value |
| Total effect | 1.21 (1.09～1.34) | **<0.001** | 1.46 (1.18～1.80) | **<0.001** |
| Direct effect | 1.19 (1.07～1.33) | **<0.001** | 1.42 (1.15～1.76) | **0.001** |
| Indirect effect | 1.01 (1.00～1.02) | **0.022** | 1.02 (1.01～1.04) | **0.022** |
| Proportion Mediated, % (95% CI) | 6.52% (0.14%～12.90%) | _ | 7.09% (0.34%～13.84%) | _ |
| FD: Food Deprivation, HR: Hazard Ratio; CI: Confidence Interval.  Adjusted for: age group, gender, residence, ethnicity, education level, marital status, maternal agricultural work before age 17, paternal agricultural work before age 17, pension insurance, medical insurance, social participation, smoking status, drinking status, BMI. | | | | |

Table S11. Causal mediation analysis of age-independent Light-BioAge acceleration in the association between childhood food deprivation and incident physical-psychological-cognitive multimorbidity (n=4,546).

| Estimated effect | Moderate FD vs No FD | | Extreme FD vs No FD | |
| --- | --- | --- | --- | --- |
|  | HR (95% CI) | *P* value | HR (95% CI) | *P* value |
| Total effect | 1.20 (1.13～1.28) | **<0.001** | 1.45 (1.27～1.64) | **<0.001** |
| Direct effect | 1.20 (1.13～1.28) | **<0.001** | 1.44 (1.27～1.63) | **<0.001** |
| Indirect effect | 1.00 (1.00～1.01) | **0.174** | 1.01 (1.00～1.01) | **0.174** |
| Proportion Mediated, % (95% CI) | 1.60% (-0.73%～3.93%) | _ | 1.74% (-0.79%～4.28%) | _ |
| FD: Food Deprivation, HR: Hazard Ratio; CI: Confidence Interval.  Adjusted for: gender, residence, ethnicity, education level, marital status, maternal agricultural work before age 17, paternal agricultural work before age 17, pension insurance, medical insurance, social participation, smoking status, drinking status, BMI. | | | | |
